# A case of simultaneous laparoscopic surgery for double cancer comprising multiple early gastric cancer and advanced sigmoid colon cancer after revascularization

**DOI:** 10.1186/s40792-021-01161-3

**Published:** 2021-04-08

**Authors:** Koichi Takiguchi, Shinji Furuya, Makoto Sudo, Kazuyoshi Hirayama, Ryo Saito, Atsushi Yamamoto, Katsutoshi Shoda, Hidenori Akaike, Naohiro Hosomura, Yoshihiko Kawaguchi, Hidetake Amemiya, Hiromichi Kawaida, Hiroshi Kono, Daisuke Ichikawa

**Affiliations:** First Department of Surgery, Faculty of Medicine, University of Yamanashi, 1110 Shimokato, Chuo, Yamanashi 409-3898 Japan

**Keywords:** Laparoscopic surgery, Double cancer, After revascularization

## Abstract

**Background:**

Traditionally, the surgery for simultaneous double cancer of the stomach and colon required a large incision to the upper and lower region of the abdomen. In this case, an artificial blood vessel was located under the skin after revascularization. Considering ischemia due to graft compression by incision retractor during laparotomy, this was difficult to do. This is a report on laparoscopic surgery for simultaneous double cancer of the stomach and colon after revascularization.

**Case presentation:**

A 69-year-old man had early gastric cancer and advanced sigmoid colon cancer. He had suffered from thromboangitis obliterans and has undergone revascularization many times due to poor blood flow in his lower limbs. He had had some artificial blood vessels inserted under the skin, confirmed by blood vessel construction image by preoperative computed tomography (CT). There was a bypass vessel from the left axillary artery to the left femoral artery under the skin of the left thoracoabdominal. In addition, there were two bypass vessels from the left external iliac artery to the right femoral artery under the skin of the lower abdomen. One of the two bypasses was occluded. In the blood flow to the intestinal tract, the inferior mesenteric artery was already occluded. Peripheral blood flow in the common iliac artery depended on blood flow from the artificial blood vessel, and blood flow from the internal iliac artery to the rectum was poor. Laparoscopic Hartmann’s operation was performed for Stage II B (UICC 8th Edition) sigmoid colon cancer. Because the blood flow in the intestinal tract on the anal side was poor, we thought that anastomosis was at a high risk for leakage. Laparoscopic total gastrectomy was also performed simultaneously for two Stage I (UICC 8th edition) gastric cancers in the cardia and body. The location of the port site and stoma was carefully determined preoperatively to prevent damage and infection to the artificial blood vessels. Minimal invasive surgery was performed using laparoscopic surgery.

**Conclusions:**

Laparoscopic surgery with small incisions is useful for patients with double cancer who need an approach to the upper and lower abdomen. Furthermore, laparoscopic surgery has less interference on graft in patients with artificial blood vessels under the skin by intraperitoneal approach.

## Background

Simultaneous double cancer of the stomach and colon is not uncommon. Preoperative screening tests for gastric cancer patients reported that 4% of patients have colorectal cancer [1]. In those cases, we operate on both the gastric cancer and colorectal cancer at the same time. Conventional laparotomy requires a large incision for simultaneous double cancer of the stomach and colon. It is a highly invasive surgery. Laparoscopic surgery is less invasive than laparotomy because the incision is smaller. Actually, it has been reported that laparoscopic surgery had a lower concentration of inflammatory cytokines in the blood than laparotomy [2]. Laparoscopic surgery is a minimal invasive surgery for simultaneous double cancer of the stomach and colon. Furthermore, laparoscopic surgery for gastric cancer and colorectal cancer has been improving in recent years. There are reports of comparative studies with laparotomy and their long-term prognosis. Gastric cancer was non-inferior to laparotomy [3]. Colorectal cancer was not proven to be non-inferior, but it was comparable [4]. Laparoscopic surgery is useful for the simultaneous double cancer of the stomach and colon because oncologically, it is safer and can be minimally invasive.

Laparoscopic surgery is also useful for patients with artificial blood vessels under the skin after revascularization. In the case of laparotomy, the artificial blood vessel may be compressed by the incision retractor. Laparoscopic surgery is one of the safest ways for patients of simultaneous double cancer of the stomach and sigmoid colon with subcutaneous artificial blood vessels after revascularization.

## Case presentation

**Fig. 1:**
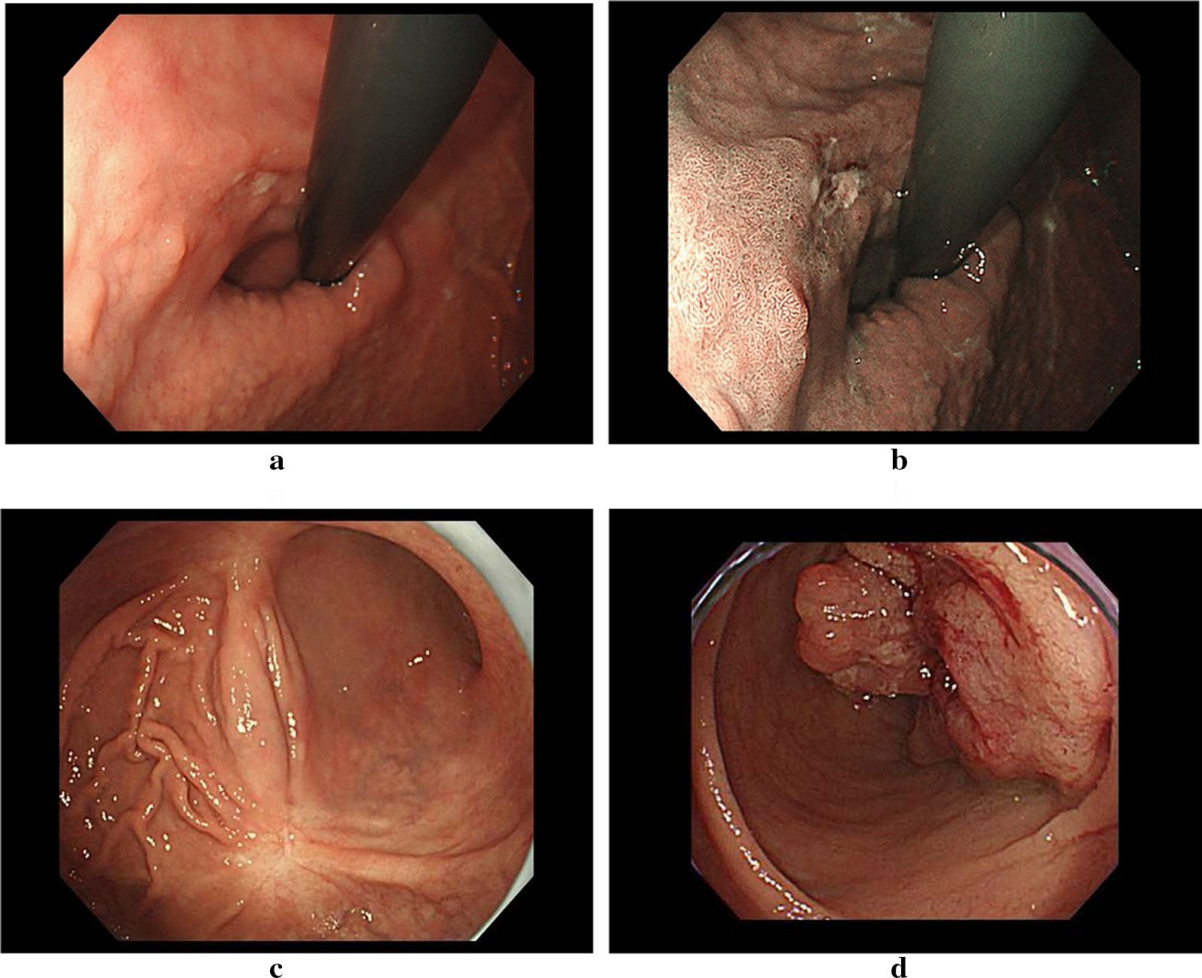
**a** Lesion in the lesser curvature of the cardia by gastrointestinal endoscopy. **b** NBI image of lesion in the lesser curvature of the cardia by gastrointestinal endoscopy. **c** Suspected gastric cancer lesion at an ulcer scar on the posterior wall of the body of the stomach. **d** Type 2 lesion in the sigmoid colon by lower gastrointestinal endoscopy

**Fig. 2:**
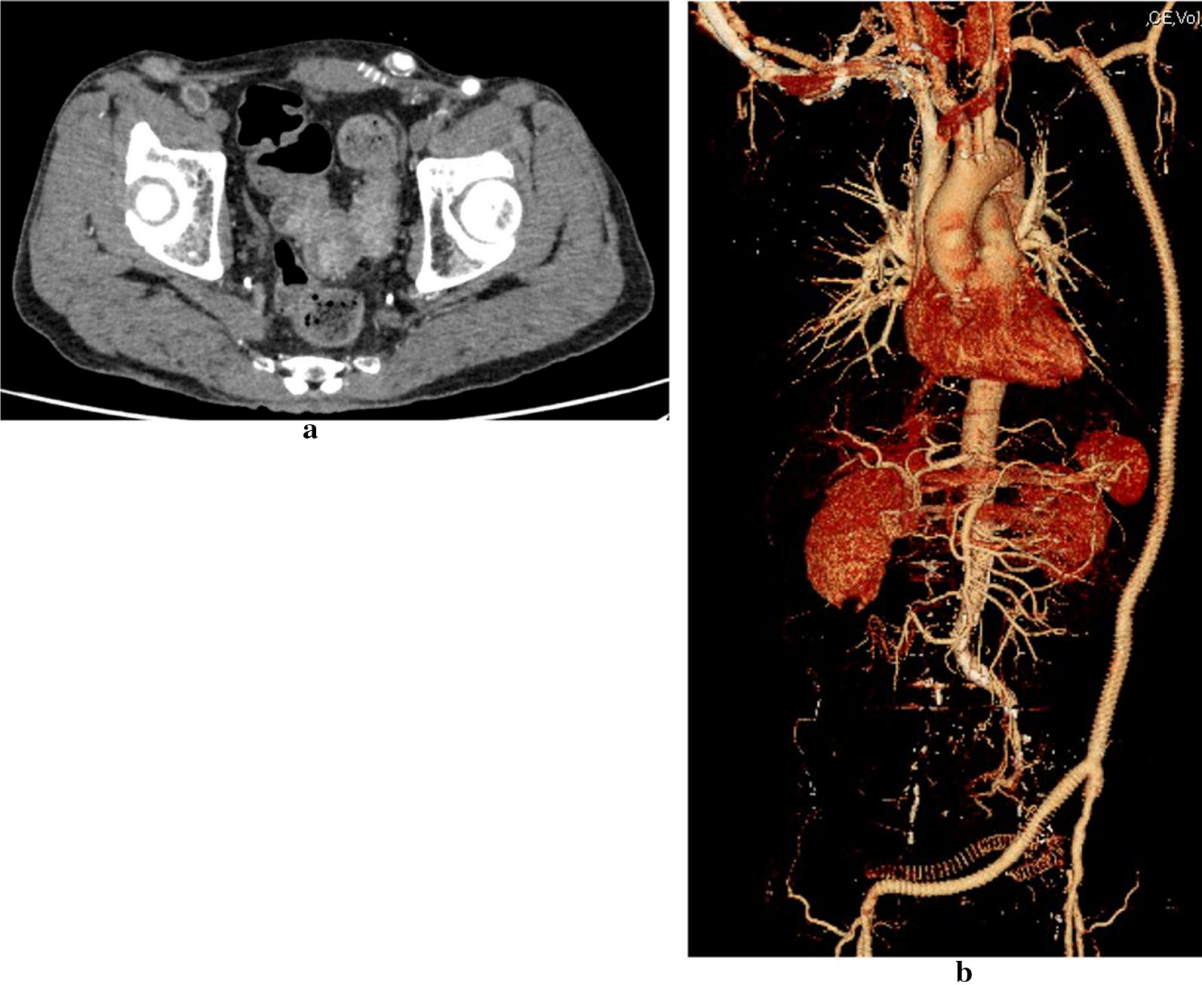
**a** Preoperative CT. The sigmoid colon had thickened walls and increased concentration of surrounding adipose tissue, but no swollen lymph nodes around the sigmoid colon lesion. **b** Preoperative blood vessel construction by 3D image analysis software SYNAPSE VINCENT® (Fujifilm, Tokyo)

A 69-year-old man was hospitalized with transient loss of consciousness due to severe anemia and melena. He had previously suffered from thromboangitis obliterans and had undergone revascularization of his lower extremities. Upper gastrointestinal endoscopy showed two lesions. One 15 mm-sized depressed lesion was present in the lesser curvature of the cardia (Fig. 1a, b). Biopsy diagnosed poor differentiated tubular adenocarcinoma. As evaluated by endoscopic ultrasound, the tumor had invaded the submucosa and surgery was necessary. The other lesion was present on the posterior wall of the body in the stomach, and a biopsy of this ulcer scar was diagnosed as suspected gastric cancer (Fig. 1c). Lower gastrointestinal endoscopy revealed a circumferential type 2 tumor in the sigmoid colon (Fig. 1d). The tumor was 38 cm from the anal verge. Biopsy revealed moderately differentiated tubular adenocarcinoma. The lumen was narrowed by this tumor, but the scope managed to pass. Preoperative computed tomography (CT) showed no swollen lymph nodes around the gastric lesions. The sigmoid colon had thickened walls and increased concentration of surrounding adipose tissue, but no swollen lymph nodes around the sigmoid colon lesion in preoperative CT (Fig. 2a). There was no distant metastasis to the gastric cancer or sigmoid colon cancer. Preoperative diagnosis was T1N0M0 Stage I (UICC 8th Edition) for two gastric cancers and T4aN0M0 Stage II B(UICC 8th Edition) for sigmoid colon cancer. The lesion in the body of the stomach was an intramucosal lesion and was singled out for endoscopic treatment, but preoperative treatment was not performed because of a lack of time. The symptoms of sigmoid colon lesions began to appear. We have had a policy of total gastrectomy for removing two gastric cancers.

**Fig. 3:**
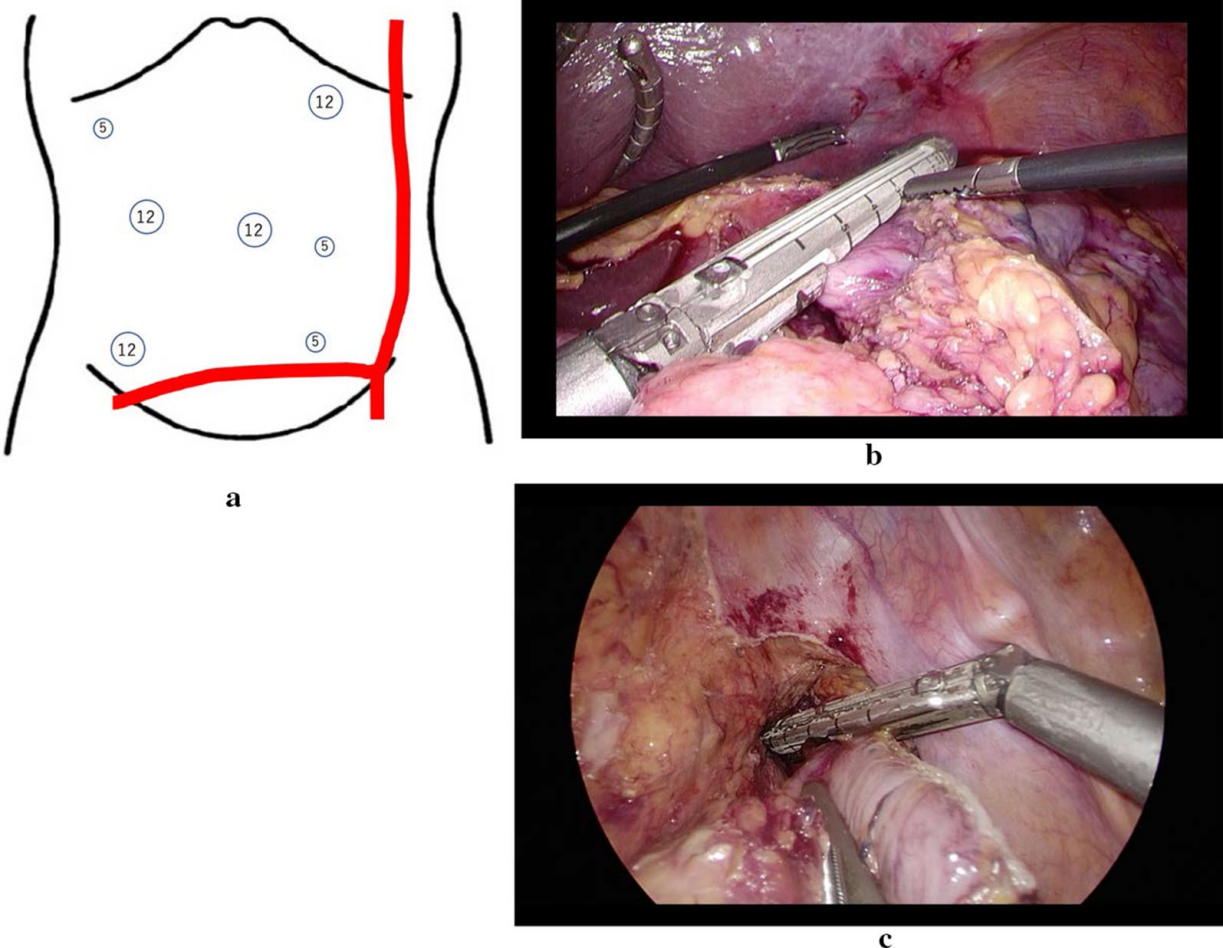
**a** Port site in this case. The port sites on the left were located more inside than usual because there are artificial blood vessels under the skin. The stoma was constructed by expanding the incision of a 5 mm port in the left middle abdomen. **b** The cardia was dissected with an automatic suture device. **c** The rectum was amputated with an automatic suture device at a distance from the tumor

Preoperative CT and blood vessel construction using 3D image analysis software SYNAPSE VINCENT® (Fujifilm, Tokyo) showed artificial blood vessels under the skin (Fig. 2b). There was a bypass vessel from the left axillary artery to the left femoral artery under the skin on the left side of thoracoabdominal region. Furthermore, there were three bypass vessels from the left external iliac artery to the right femoral artery under the skin of the lower abdomen. Two of the three bypasses were occluded. In the blood flow to the intestinal tract, the inferior mesenteric artery was already occluded. Peripheral blood flow in the common iliac artery depended on blood flow from the artificial blood vessel, and blood flow from the internal iliac artery to the rectum was poor. Blood flow in the anal intestinal tract after excision seemed to be poor, and anastomosis was judged to be at a high risk of leakage. We have a policy of using laparoscopic Hartmann’s surgery. Port and stoma sites were decided by heeding the position of the artificial blood vessels. The port sites on the left were located more inside than usual. (Fig. 3a) First, the stomach was resected, and the stump was evaluated pathologically. (Fig. 3b) There was no tumor residue on the stump. Next, the sigmoid colon was resected before reconstruction (Fig. 3c). And then stomach reconstruction was performed by Roux-en-Y reconstruction. The jejunum was lifted anterior transverse colon pathway. Finally, the stoma was made in the lower left abdomen. The operation time was 11 h and 24 min. The blood loss volume was 74 ml. Postoperatively, fever was observed at 9 days after the operation, and CT showed pancreatic fistula (grade B). The patient improved with antibiotics. He was discharged at 21 days after the operation. The pathological diagnosis showed that there was another lesion in the stomach. It was unknown before the operation. In the end, there were three lesions. One was a submucosal invasion, and the others were intramucosal cancers. All were Stage I. Sigmoid colon cancer was T3N0M0 Stage II A. Two years have passed since the operation and there has been no recurrence.

## Discussion

One of the advantages of laparoscopic surgery is small incisions. The advantage is significant for simultaneous double cancers like in our case. We can approach from the upper or the lower abdomen with small incisions. The minimal invasiveness of laparoscopic surgery is useful. In addition, this case had artificial blood vessels under the skin. In the case of laparotomy, the graft may be compressed because the incision is widened by the retractor. That would lead to poor blood flow in the lower limbs. Laparoscopic surgery has less invasiveness on subcutaneous grafts due to smaller incisions. At the time of surgery for gastric cancer and colorectal cancer, the first issue is the location of the port site. Laparoscopic surgery for gastric cancer is usually performed by five ports, and laparoscopic surgery for colorectal cancer is also performed by five. The umbilical camera port and left and right mid-abdominal ports can be used as common ports for gastric cancer and left colorectal cancer to perform simultaneous surgery. We performed simultaneous resection of gastric cancer and sigmoid colon cancer by seven ports. The mid-abdominal port should be inserted in an effective position for both the upper and lower abdomen. A 12 mm port was used as the port for inserting the suture device. Matsui et al. reported that the stomach and sigmoid colon were resected at seven ports [5]. They added one in the epigastric and one in the lower abdomen to the usual five ports. In cases with a narrow abdomen, the policy of adding to the usual five ports as in their case may reduce the number of ports.

Regarding the procedure of resection and reconstruction, they recommended that resection and reconstruction of the stomach be performed before the colorectal resection. The reason was to reduce blood loss from the resected specimen. With the current reconstruction method, the stump is closed once with a suture, so there is little blood loss. Therefore, resection and reconstruction should be considered separately. If the other resection is performed after reconstruction, the resection operation may strain the anastomotic site. The order of resection is arbitrary. However, it should be performed on lesions that are uncertain to be resected or not, or lesions that require evaluation of the stump. Regarding reconstruction, Roux-en-Y reconstruction should be done last. The reason is that the positional relationship with the colon is important. Since this case was a stoma construction, the stoma construction was performed last.

In this case, it was judged that the blood flow from the internal iliac artery to the rectum was poor before the operation, and anastomosis was not performed. In recent years, it has been reported that intraoperative ICG blood flow measurement may reduce anastomotic leakage [6]. If intraoperative ICG measurement was possible at that time, this case might have been anastomosed. We are looking forward to the results of a large-scale randomized controlled trial of an ICG blood flow assessment.

## Conclusions

Laparoscopic surgery for a patient with simultaneous double cancer of the stomach and sigmoid colon after revascularization was performed. Laparoscopic surgery is even more useful than laparotomy for higher risk surgery due to its minimal invasive nature.

## Data Availability

All data generated or analyzed during this article are included in this published article.

## Abbreviations

CT: Computed tomography
UICC: Union for International Cancer Control
ICG: Indocyanine green
NBI: Narrow band imaging
